# Antibiotic resistance prediction for *Mycobacterium tuberculosis* from genome sequence data with Mykrobe

**DOI:** 10.12688/wellcomeopenres.15603.1

**Published:** 2019-12-02

**Authors:** Martin Hunt, Phelim Bradley, Simon Grandjean Lapierre, Simon Heys, Mark Thomsit, Michael B. Hall, Kerri M. Malone, Penelope Wintringer, Timothy M. Walker, Daniela M. Cirillo, Iñaki Comas, Maha R. Farhat, Phillip Fowler, Jennifer Gardy, Nazir Ismail, Thomas A. Kohl, Vanessa Mathys, Matthias Merker, Stefan Niemann, Shaheed Vally Omar, Vitali Sintchenko, Grace Smith, Dick van Soolingen, Philip Supply, Sabira Tahseen, Mark Wilcox, Irena Arandjelovic, Tim E. A. Peto, Derrick W. Crook, Zamin Iqbal

**Affiliations:** 1 European Bioinformatics Institute, Cambridge, UK; 2 Nuffield Department of Medicine, University of Oxford, Oxford, UK; 3 Centre de Recherche du Centre Hospitalier de l'Universite de Montreal, Montreal, Canada; 4 Infectiology & immunology department, Universite de Montreal Microbiology, Montreal, Canada; 5 Oxford University Clinical Research Unit, Ho Chi Minh City, Vietnam; 6 Emerging Bacterial Pathogens Unit, WHO collaborating Centre and TB Supranational Reference laboratory, IRCCS San Raffaele Scientific institute, Milan, Italy; 7 Instituto de Biomedicina de Valencia (IBV-CSIC), Valencia, Spain; 8 FISABIO Public Health, Valencia, Spain; 9 CIBER in Epidemiology and Public Health, Madrid, Spain; 10 Harvard Medical School, Boston, USA; 11 British Columbia Centre for Disease Control, Vancouver, Canada; 12 Bill and Melinda Gates Foundation, Seattle, USA; 13 National Institute for Communicable Diseases (NICD), Johannesburg, South Africa; 14 Forschungszentrum Borstel, Leibniz Lungenzentrum, Borstel, Germany; 15 Unit Bacterial Diseases Service, Infectious Diseases in Humans, Sciensano, Brussels, Belgium; 16 German Center for Infection Research, Borstel Site, Borstel, Germany; 17 Centre for Infectious Diseases and Microbiology - Public Health, University of Sydney, Sydney, Australia; 18 National Mycobacterial Reference Service, Public Health England Public Health Laboratory, Birmingham, UK; 19 National Institute for Public Health and the Environment (RIVM), Bilthoven, The Netherlands; 20 Univ. Lille, CNRS, Inserm, CHU Lille, Institut Pasteur de Lille, U1019 - UMR 8204 - CIIL - Centre d'Infection et d'Immunite de Lille, Lille, France; 21 National TB Reference Laboratory, National TB control Program, Islamabad, Pakistan; 22 Leeds Teaching Hospital NHS Trust, Leeds, UK; 23 University of Leeds, Leeds, UK; 24 Faculty of Medicine, Institute of Microbiology and Immunology, Belgrade, Serbia; 25 National Infection Service, Public Health England, UK

**Keywords:** Antimicrobial resistance, tuberculosis, diagnostic, nanopore, whole genome sequencing, antibiotic treatment

## Abstract

Two billion people are infected with *Mycobacterium tuberculosis*, leading to 10 million new cases of active tuberculosis and 1.5 million deaths annually. Universal access to drug susceptibility testing (DST) has become a World Health Organization priority. We previously developed a software tool, *Mykrobe predictor*, which provided offline species identification and drug resistance predictions for *M. tuberculosis *from whole genome sequencing (WGS) data. Performance was insufficient to support the use of WGS as an alternative to conventional phenotype-based DST, due to mutation catalogue limitations.

Here we present a new tool, *Mykrobe*, which provides the same functionality based on a new software implementation. Improvements include i) an updated mutation catalogue giving greater sensitivity to detect pyrazinamide resistance, ii) support for user-defined resistance catalogues, iii) improved identification of non-tuberculous mycobacterial species, and iv) an updated statistical model for Oxford Nanopore Technologies sequencing data. *Mykrobe* is released under MIT license at https://github.com/mykrobe-tools/mykrobe. We incorporate mutation catalogues from the CRyPTIC consortium et al. (2018) and from Walker et al. (2015), and make improvements based on performance on an initial set of 3206 and an independent set of 5845 *M. tuberculosis* Illumina sequences. To give estimates of error rates, we use a prospectively collected dataset of 4362 *M. tuberculosis isolates*. Using culture based DST as the reference, we estimate *Mykrobe* to be 100%, 95%, 82%, 99% sensitive and 99%, 100%, 99%, 99% specific for rifampicin, isoniazid, pyrazinamide and ethambutol resistance prediction respectively. We benchmark against four other tools on 10207 (=5845+4362) samples, and also show that *Mykrobe* gives concordant results with nanopore data.

We measure the ability of *Mykrobe*-based DST to guide personalized therapeutic regimen design in the context of complex drug susceptibility profiles, showing 94% concordance of implied regimen with that driven by phenotypic DST, higher than all other benchmarked tools.

## Introduction

**Figure 1.**
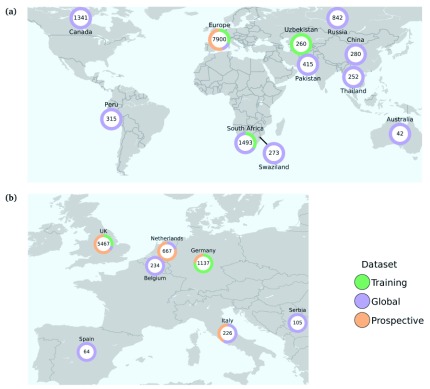
Global ( **a**) and European ( **b**) origin of samples included in this study. Numbers represent the total numbers of samples from each region or country. Colours represent the proportion in each study dataset (see Source data sample_data.tsv for complete details ^29^).

The software tool *Mykrobe predictor* ^1^, released in 2015, identified isolates to the species level and predicted the drug susceptibility testing (DST) profile of *Staphylococcus aureus* and *Mycobacterium tuberculosis* directly from genomic sequencing data. *Mykrobe predictor* was developed to address four needs. First, it provided robust genotyping of single nucleotide polymorphisms (SNPs), insertions and deletions (indel) and gene alleles, independently of the location of mutations (where "location" is used both in the sense of coordinate, and in the sense of chromosomal versus plasmid). It also did this with error rates that did not depend on the phylogenetic position of the isolate of interest, a characteristic of all reference-genome methods ^2^. We did this by building a de Bruijn “genome graph” representation of known variation in the species, incorporating both resistant and susceptible alleles, and also nearby variation that might affect *k*-mer matching (equivalent to neighbouring mutations affecting a PCR probe (see Figure 1 of 1)). Second, it identified target species, with prior knowledge of likely contaminants, and prevented misdiagnosis of resistance due to shared elements. Third, it predicted phenotype from genotype, including from low (within-isolate) frequency alleles. Fourth, it integrated these functionalities in a fast, lightweight, internet-free and user-friendly platform.

In that initial publication ^1^, for *M. tuberculosis*, the drug resistance conferring mutation panel was based on first and second generation line probe assays (LPA) (HAIN Lifesciences, Nehren, Germany) resulting in low sensitivity but high specificity. We also showed that detecting minor populations of resistant alleles improved the ability to predict resistance for some second-line drugs (amikacin, capreomycin, ofloxacin), although the low number of samples with associated second-line phenotypic data left this analysis underpowered, and needing replication.

There were a number of improvements we wished to make over the 2015 *Mykrobe predictor* codebase. First, we wanted a cleaner codebase. Second we wanted users to be able to specify their own resistance catalogue. The latest *Mykrobe predictor* catalogue, updated since publication, was based on Walker *et al.* ^3^. Walker *et al.* derived this catalogue by analysing the same 3500 samples which were used in the initial *Mykrobe predictor* performance analysis ^1^ - thus no independent test or validation dataset were available to properly estimate error rates. Finally, we wanted to further develop the support for nanopore data which had been improved by the use of a slightly different statistical model ^4^, but only tested on *N* = 5 replicates of a single strain of *M. bovis* substrain BCG.

A number of other tools for detection of resistance-associated alleles have been released: ABRicate (Seemann), ARIBA ^5^, ARGs-OAP ^6^, ARG-ANNOT ^7^, CASTB ^8^, KvarQ ^9^, MTBseq ^10^, PhyResSe ^11^, RAST ^12^, ResFinder ^13^, RGI ^14^, SRST2 ^15^, SSTAR ^16^, and TB-Profiler ^17^. The majority look for gene presence, primarily in *enterobacteriaceae*, but some also look for SNP and indel mutations. In particular, SRST2 which maps reads to a panel of alleles (tested on *S. aureus*, *Streptococcus pneumoniae*, *Salmonella enterica* serovar Typhimurium, *Shigella sonnei*, *Enterococcus faecium*, *Listeria monocytogenes* and *Klebsiella pneumoniae*), and ARIBA which performs local assembly (tested on *E. faecium*, *S. sonnei* and *Neisseria gonorrhoeae*). In the specific case of *M. tuberculosis*, where there is very little recombination, resistance prediction is dominated by the ability to correctly genotype SNPs and indels. Various tools have been developed to achieve this. These include the web-tool PhyResSE ^11^, KvarQ ^9^ which uses *k*-mers, and TB-Profiler ^17^ which uses mapping; earlier versions were benchmarked in 18. Another tool, MTBseq ^10^ is a general mapping-based WGS pipeline for *M. tuberculosis* which can in particular be used to predict resistance. Our expectation was that for *M. tuberculosis*, the resistance panel would be the key determinant of sensitivity and specificity, as was previously demonstrated for *S. aureus* ^19^. We confirm this below.

In a recent study on resistance to first-line tuberculosis (TB) drugs (rifampicin, isoniazid, ethambutol and pyrazinamide) ^20^ the authors show, for the first time, that sequencing-based prediction of susceptibility is accurate enough for clinical use. They classify resistance-associated gene mutations as i) resistance-causing mutations, ii) definitely not resistance-causing mutations, or iii) unknown significance mutations. Their resistance mutation catalogue included variants from a systematic review from Miotto *et al.* ^21^ and some new *pncA* mutations from 22. It also characterised all frame-shifts in *pncA* and *katG* as causing resistance to pyrazinamide or isoniazid respectively. Using phenotyping as gold standard, they measured performance characteristics of WGS-based DST, including sensitivity and specificity, on the samples for which a prediction can be made with high confidence. They propose a workflow where a sample is sequenced, and if an "unknown significance" mutation is seen, then no resistance/susceptibility prediction is made, but the sample is instead referred for phenotyping. This provides a means whereby healthcare systems can leverage the advantages of WGS for rapid and comprehensive DST, be informed when a given sample is pushing against the limits of our knowledge, and revert to slower but well-understood and trusted phenotyping in order to provide clinicians with reliable results, and prospectively enrich available databases.

In this study we evaluate our new lightweight offline species-identification and resistance prediction tool, named simply *Mykrobe* ^23^. The species-identification methodology is unchanged since ^1^, but after publication *Mykrobe predictor* was heavily evaluated by Public Health England, and in order to pass their acceptance criteria, we improved our species-informative probes. We report the results of applying these prospectively at PHE below, but this study primarily concerns improvements in resistance prediction. We first combine the resistance catalogue from The CRyPTIC Consortium *et al.* ^20^ with the pre-existing second-line drug resistance catalogue from Walker *et al.* ^3^. We treat this catalogue as our first candidate catalogue and improve it in an iterative process, evaluating it on successive datasets.

We benchmark *Mykrobe* against a range of other tools: ARIBA (using the same catalogue as *Mykrobe*, as a control), and also KvarQ, MTBseq, and TB-profiler using their inbuilt catalogues. In contrast to the approach introduced in 20, *Mykrobe* has no concept of "unknown significance", predicting susceptibility if there is no known resistance mutation. We quantify the trade-offs between these approaches.

The World Health Organisation (WHO) publishes guidelines for TB treatment including drug susceptible, single drug-resistant, multidrug-resistant (MDR-TB) and extensively drug-resistant TB (XDR-TB) ^24– 27^. These guidelines are regularly reviewed and updated to appropriately reflect, and adapt to, the evolution of *M. tuberculosis* resistance and the availability of novel TB drugs, whether new or re-purposed. Based on latest WHO recommendations, we simulate the ability of *Mykrobe* to guide personalized therapeutic regimen design and compare the *Mykrobe*-inferred regimen against that implied by phenotypic testing. We find that *Mykrobe* compares favourably with the gold-standard approach and is superior to the other tools we benchmark. We believe that this is a novel and important metric for the evaluation of an *in silico* DST tool.

One ongoing challenge for the development of *in silico* DST, is that only limited phenotype and sequence data are shared openly for reuse and validation. We address both of these problems in this paper. First, we make available all our underlying data both in public and in easily-reusable text format. Second, we chose to publish in this journal specifically because we can update the paper as the catalogue is modified and when competitor tools are updated, to give current results pointing to the latest underlying data.

## Results

### Species identification

*Mykrobe predictor* was evaluated extensively by Public Health England (PHE) for species identification of *Mycobacteria* in 2016 and a need to improve on the identification of non-tuberculous Mycobacteria (NTMs) was identified. As a result, a new set of probes was built by augmenting the initial training set to 1018 samples, building a pooled de Bruijn graph, and searching for species informative contigs (method described in 1) to use as probes. These were evaluated in a PHE laboratory where all mycobacterial samples were prospectively sequenced over the course of one year, and the results published ^28^: of 1902 samples for which Line Probe Assay (LPA) testing had identified a clinically important mycobacterium and whole genome sequencing data was also available, 1825 (96%) were correctly identified to the species level by *Mykrobe* (treating the LPA as gold standard). *Mykrobe* ^23^ incorrectly identified 33 isolates as a different species within the same complex (6 MTBC, 3 *M. abscessus* complex, 7 *M. avium* complex, and 17 *M. fortuitum* complex isolates). We have adopted the same probes in *Mykrobe*, and no further evaluation has been done in this study.

### Drug resistance prediction

**Data.** We re-use the sequencing and phenotype data from 1, 54 and 51; the latter included only first-line phenotypes, so in this study we augment that data with second-line phenotype data. We also remove in advance those samples which were identified as likely sample-swaps in 51. We split the samples into three disjoint datasets:

- Training set: *N* = 3206 isolates, from 1)
- Prospective set: *N* = 4362 isolates from 20; specifically these were the only isolates that were sampled prospectively (from Italy, Germany, the Netherlands and the UK) to enable realistic error-estimates.
- Global set: *N* = 5845, the remaining isolates from 20.

See Figure 1 and *Source data* sample_data.tsv ^29^ for a per-country breakdown of the datasets.

**Figure 2.**
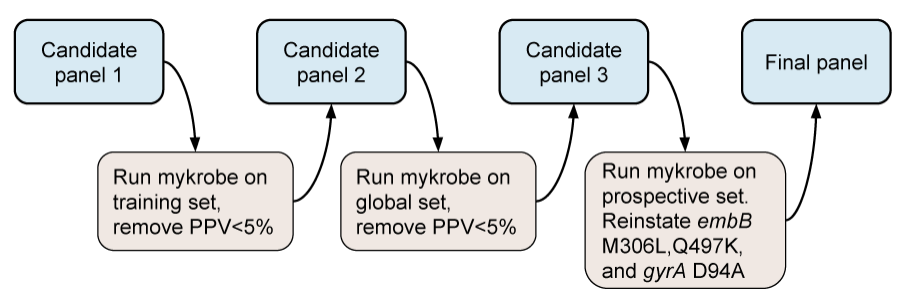
Process for developing the new *Mykrobe* variant panel. See main text for details.

**Initial development of resistance panel.** We started with a mutation catalogue (candidate panel 1, CP1) built by combining those from 20 for first-line drugs and 3 for second-line drugs. An iterative process was used to remove variants from the panel after running *Mykrobe* on each dataset (see Methods and Figure 2 for details). First, CP1 was evaluated on the Training set. This resulted in the removal of six variants from the panel which had positive predictive value less than 5%, producing candidate panel 2, CP2 (see Methods for details). CP1 is given in *Source data* panel.CP1.tsv ^30^, panel.CP1.json ^31^, and the six variants removed from CP1 to generate CP2 are in *Source data* removed_variants.tsv ^32^. We then measured the performance of *Mykrobe* with this panel on the large Global dataset.

**Figure 3.**
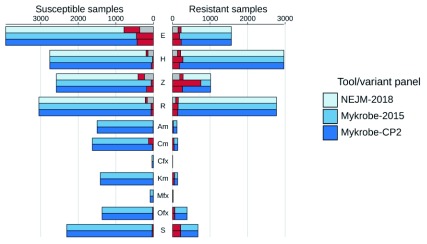
Comparison of NEJM-2018 (results from 20), *Mykrobe* using the Walker-2015 panel and *Mykrobe* using candidate panel 2 (CP2) on the Global dataset. Counts of susceptible samples are shown on the left, broken down for each tool/panel into those correctly identified as susceptible (in the colour of the tool/panel), those falsely identified as resistant coloured red and no-calls coloured grey. Similarly, resistant samples are shown on the right, with those correctly identified as resistant coloured by the tool/panel, those falsely identified as susceptible in red, and no-calls in grey. Note that second-line drugs were not considered in NEJM-2018, and so are not shown. E, ethambutol; H, isoniazid; Z, pyrazinamide; R, rifampicin; Am, amikacin; Cm, capreomycin; Cfx, ciprofloxacin; Km, kanamycin; Mfx, moxifloxacin; Ofx, ofloxacin; S, streptomycin.

**Resistance prediction performance on Global dataset.** We show in Figure 3 a comparison of three sets of predictions on the Global dataset: those from 20, those from *Mykrobe* using the panel ("Walker-2015") used by our previous software *Mykrobe predictor*, and those from *Mykrobe* using CP2 (complete results are in *Extended data* file accuracy_stats.tsv ^33^). The greatest difference in performance based on Walker-2015 and CP2 is in pyrazinamide, where sensitivity increases from 25% in Walker-2015 to 74% in CP2, at the cost of specificity falling from 98% to 94%. Numerous rare variants were the source of reduced pyrazinamide specificity in CP2, most of which also cause true-positives ( *Extended data* file variant_counts.tsv ^34^). However, 21 of these variants resulted in 29 false-positives and no true-positives and were therefore removed from CP2 to make CP3. We did however notice that of these 21, 16 were in the resistance catalogue from 22, which provides direct experimental evidence for their impact, and so we may reinstate them in future given further data.

The difference between Walker-2015 (86% sensitivity, 89% specificity) and CP2 (84% sensitivity, 89% specificity) for ethambutol is explained by two variants in the *embB* gene. The first site, M306, is included in all panels. However, in Walker-2015 it is present as any non-synonymous change, whereas in NEJM-2018 (and hence CP1 and CP2) only the more specific M306I and M306V are included. The variant M306L (in all samples found as ATG changed to CTG) causes 27 correct resistant calls, but also 14 false-positives. The second site is Q497K, which is in both Walker-2015 and CP1, causes 15 true-positive and 5 false-positive calls on the Global dataset, but was excluded from CP2 because its only contribution was 3 false-positives on the Training dataset.

In terms of bottom-line statistics on this, our largest dataset containing the widest range of resistance alleles, Very Major Error rate (VME, missed resistance) for the first-line drugs was 4.9%, 6.2%, 14.7%, 26.0% for rifampicin, isoniazid, ethambutol, pyrazinamide respectively, with corresponding negative predictive values of 95.7%, 93.6%, 93.8%, 90.1%. The Major Error rate (false resistance prediction) was: 2.0%, 2.0%, 11.0%, 6.9% respectively. These results can be seen in Table 1b. We discuss below how the approach taken in 20 improved on this VME despite using essentially the same catalogue.

**Table 1.** Performance of *Mykrobe* during development of the variant panel. TP, number of phenotypically resistant samples that correctly identified as resistant ("true positives"); FP, number of phenotypically susceptible samples which are falsely identified as resistant ("false positives"); TN, number of phenotypically susceptible samples that are correctly identified as susceptible ("true negatives"); FN, number of phenotypically resistant samples that are incorrectly identified as susceptible ("false negative"); VME, very major error rate (false-negative rate); ME, major error rate (false-positive rate); PPV, positive predictive value; NPV, negative predictive value. 95% binomial confidence intervals calculated using the Wilson score interval.

(a) Candidate panel 1, Training dataset								
Drug	TP	FP	TN	FN	VME (95% CI)	ME (95% CI)	PPV (95% CI)	NPV (95% CI)
Ethambutol	209	121	2807	22	9.52(6.4-14.0%)	4.13(3.5-4.9%)	63.33(58.0-68.3%)	99.22(98.8-99.5%)
Isoniazid	564	32	2538	38	6.31(4.6-8.5%)	1.25(0.9-1.8%)	94.63(92.5-96.2%)	98.52(98.0-98.9%)
Rifampicin	373	40	2714	11	2.86(1.6-5.1%)	1.45(1.1-2.0%)	90.31(87.1-92.8%)	99.6(99.3-99.8%)
Amikacin	56	3	702	8	12.5(6.5-22.8%)	0.43(0.1-1.2%)	94.92(86.1-98.3%)	98.87(97.8-99.4%)
Capreomycin	51	36	669	10	16.39(9.2-27.6%)	5.11(3.7-7.0%)	58.62(48.1-68.4%)	98.53(97.3-99.2%)
Ciprofloxacin	19	4	243	0	0.0(0.0-16.8%)	1.62(0.6-4.1%)	82.61(62.9-93.0%)	100.0(98.4-100.0%)
Kanamycin	11	1	528	6	35.29(17.3-58.7%)	0.19(0.0-1.1%)	91.67(64.6-98.5%)	98.88(97.6-99.5%)
Moxifloxacin	17	5	549	4	19.05(7.7-40.0%)	0.9(0.4-2.1%)	77.27(56.6-89.9%)	99.28(98.2-99.7%)
Ofloxacin	19	4	763	6	24.0(11.5-43.4%)	0.52(0.2-1.3%)	82.61(62.9-93.0%)	99.22(98.3-99.6%)
Streptomycin	344	18	1355	39	10.18(7.5-13.6%)	1.31(0.8-2.1%)	95.03(92.3-96.8%)	97.2(96.2-97.9%)
(b) Candidate panel 2, Global dataset								
Drug	TP	FP	TN	FN	VME (95% CI)	ME (95% CI)	PPV (95% CI)	NPV (95% CI)
Ethambutol	1338	434	3499	230	14.67(13.0-16.5%)	11.03(10.1-12.1%)	75.51(73.5-77.5%)	93.83(93.0-94.6%)
Isoniazid	2781	54	2704	184	6.21(5.4-7.1%)	1.96(1.5-2.5%)	98.1(97.5-98.5%)	93.63(92.7-94.5%)
Pyrazinamide	750	177	2408	264	26.04(23.4-28.8%)	6.85(5.9-7.9%)	80.91(78.3-83.3%)	90.12(88.9-91.2%)
Rifampicin	2634	60	2990	136	4.91(4.2-5.8%)	1.97(1.5-2.5%)	97.77(97.1-98.3%)	95.65(94.9-96.3%)
Amikacin	100	5	1495	21	17.36(11.6-25.1%)	0.33(0.1-0.8%)	95.24(89.3-97.9%)	98.61(97.9-99.1%)
Capreomycin	97	26	1600	42	30.22(23.2-38.3%)	1.6(1.1-2.3%)	78.86(70.8-85.1%)	97.44(96.6-98.1%)
Ciprofloxacin	1	4	33	4	80.0(37.6-96.4%)	10.81(4.3-24.7%)	20.0(3.6-62.4%)	89.19(75.3-95.7%)
Kanamycin	88	3	1410	47	34.81(27.3-43.2%)	0.21(0.1-0.6%)	96.7(90.8-98.9%)	96.77(95.7-97.6%)
Moxifloxacin	14	1	85	3	17.65(6.2-41.0%)	1.16(0.2-6.3%)	93.33(70.2-98.8%)	96.59(90.5-98.8%)
Ofloxacin	329	19	1348	59	15.21(12.0-19.1%)	1.39(0.9-2.2%)	94.54(91.6-96.5%)	95.81(94.6-96.7%)
Streptomycin	462	32	2276	217	31.96(28.6-35.6%)	1.39(1.0-2.0%)	93.52(91.0-95.4%)	91.3(90.1-92.3%)
(c) Candidate panel 3, prospective dataset								
Drug	TP	FP	TN	FN	VME (95% CI)	ME (95% CI)	PPV (95% CI)	NPV (95% CI)
Ethambutol	71	48	4171	1	1.39(0.2-7.5%)	1.14(0.9-1.5%)	59.66(50.7-68.0%)	99.98(99.9-100.0%)
Isoniazid	312	19	3931	17	5.17(3.3-8.1%)	0.48(0.3-0.7%)	94.26(91.2-96.3%)	99.57(99.3-99.7%)
Pyrazinamide	102	23	4047	23	18.4(12.6-26.1%)	0.57(0.4-0.8%)	81.6(73.9-87.4%)	99.43(99.2-99.6%)
Rifampicin	133	35	4140	0	0.0(0.0-2.8%)	0.84(0.6-1.2%)	79.17(72.4-84.6%)	100.0(99.9-100.0%)
Amikacin	3	0	59	1	25.0(4.6-69.9%)	0.0(0.0-6.1%)	100.0(43.9-100.0%)	98.33(91.2-99.7%)
Capreomycin	3	0	59	0	0.0(0.0-56.1%)	0.0(0.0-6.1%)	100.0(43.9-100.0%)	100.0(93.9-100.0%)
Ciprofloxacin	30	3	199	19	38.78(26.4-52.7%)	1.49(0.5-4.3%)	90.91(76.4-96.9%)	91.28(86.8-94.3%)
Kanamycin	4	0	54	0	0.0(0.0-49.0%)	0.0(0.0-6.6%)	100.0(51.0-100.0%)	100.0(93.4-100.0%)
Moxifloxacin	19	3	41	1	5.0(0.9-23.6%)	6.82(2.3-18.2%)	86.36(66.7-95.2%)	97.62(87.7-99.6%)
Ofloxacin	21	3	42	1	4.55(0.8-21.8%)	6.67(2.3-17.8%)	87.5(69.0-95.7%)	97.67(87.9-99.6%)
Streptomycin	36	16	685	21	36.84(25.5-49.8%)	2.28(1.4-3.7%)	69.23(55.7-80.1%)	97.03(95.5-98.0%)

**Figure 4.**
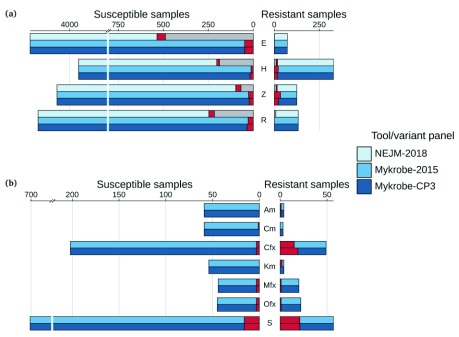
Comparison of NEJM-2018, Walker-2015 and candidate panel 3 (CP3) on the Prospective dataset for ( **a**) first-line drugs and ( **b**) second-line drugs. Counts of susceptible samples are shown on the left, broken down for each tool/panel into those correctly identified as susceptible (in the colour of the tool/panel), those falsely identified as resistant coloured red and no-calls coloured grey. Similarly, resistant samples are shown on the right, with those correctly identified as resistant coloured by the tool/panel, those falsely identified as susceptible in red, and no-calls in grey. Note that second-line drugs were not considered in NEJM-2018. E, ethambutol; H, isoniazid; Z, pyrazinamide; R, rifampicin; Am, amikacin; Cm, capreomycin; Cfx, ciprofloxacin; Km, kanamycin; Mfx, moxifloxacin; Ofx, ofloxacin; S, streptomycin.

**Resistance prediction performance on Prospective dataset** Different resistance mutations occur at varying frequencies across the world. Therefore, in order for an error-rate estimate to be generalisable into clinical practise, the underlying data needs to be sampled in a way that is representative of prevalence somewhere. We used the Prospective dataset to get realistic estimates of error rates in the (low burden) countries from which these data were sampled. We show in Figure 4 a comparison of the predictions for that dataset from 20, *Mykrobe* using the old *Mykrobe predictor* panel (Mykrobe-2015) ^3^, and *Mykrobe* with CP3.

Sensitivity, specificity, and error rates are shown in Table 1c. Very Major Error rate (missed resistance) for the first-line drugs was 0.0%, 5.2%, 1.4%, 18.4% for rifampicin, isoniazid, ethambutol, pyrazinamide respectively, with corresponding negative predictive values of 100.0%, 99.6%, 100.0%, 99.4%. Thus, in this prevalence setting, *Mykrobe* predicting susceptibility to the four first-line drugs would meet clinical requirements. The Major Error rate (false resistance prediction) was also low: 0.8%/0.5%/1.1%/0.6% respectively.

We noted that ciprofloxacin sensitivity was lower for CP3, compared to Walker-2015. This was caused by the (standard line probe assay) variant D94A in the *gyrA* gene having been removed from CP2 to make CP3, because it caused one false-positive and no true-positive calls on the Global data set. However, on the Prospective data set its inclusion causes four true-positives and no false-positives.

Based on the above results, the three variants embB M306L and Q497K, and gyrA D94A were added to CP3, to make the final variant panel. These final variants are given in *Source data* panel.final.tsv ^35^, panel.final.json ^36^, which are the same as those used by default in the new *Mykrobe* release version 0.7.0. The accuracy statistics of candidate panels 1, 2, and 3 on the Training, Global and Prospective datasets are given in Table 1 (a), (b), and (c) respectively. The results of the final release panel on each of the datasets are in Table 2.

**Table 2.** Accuracy statistics of *Mykrobe* using the final panel on (a) Training, (b) Global, and (c) Prospective datasets. TP, true-positive; FP, false-positive; TN, true-negative; FN, false-negative; VME, very major error rate (false-negative rate); ME, major error rate (false-positive rate); PPV, positive predictive value; NPV, negative predictive value. 95% binomial confidence intervals calculated using the Wilson score interval.

(a) Training dataset								
Drug	TP	FP	TN	FN	VME (95% CI)	ME (95% CI)	PPV (95% CI)	NPV (95% CI)
Ethambutol	209	125	2803	22	9.52(6.4-14.0%)	4.27(3.6-5.1%)	62.57(57.3-67.6%)	99.22(98.8-99.5%)
Isoniazid	564	32	2538	38	6.31(4.6-8.5%)	1.25(0.9-1.8%)	94.63(92.5-96.2%)	98.52(98.0-98.9%)
Rifampicin	373	40	2714	11	2.86(1.6-5.1%)	1.45(1.1-2.0%)	90.31(87.1-92.8%)	99.6(99.3-99.8%)
Amikacin	55	1	704	9	14.06(7.6-24.6%)	0.14(0.0-0.8%)	98.21(90.6-99.7%)	98.74(97.6-99.3%)
Capreomycin	49	10	695	12	19.67(11.6-31.3%)	1.42(0.8-2.6%)	83.05(71.5-90.5%)	98.3(97.1-99.0%)
Ciprofloxacin	19	4	243	0	0.0(0.0-16.8%)	1.62(0.6-4.1%)	82.61(62.9-93.0%)	100.0(98.4-100.0%)
Kanamycin	11	0	529	6	35.29(17.3-58.7%)	0.0(0.0-0.7%)	100.0(74.1-100.0%)	98.88(97.6-99.5%)
Moxifloxacin	17	4	550	4	19.05(7.7-40.0%)	0.72(0.3-1.8%)	80.95(60.0-92.3%)	99.28(98.2-99.7%)
Ofloxacin	19	3	764	6	24.0(11.5-43.4%)	0.39(0.1-1.1%)	86.36(66.7-95.2%)	99.22(98.3-99.6%)
Streptomycin	340	16	1357	43	11.23(8.4-14.8%)	1.17(0.7-1.9%)	95.51(92.8-97.2%)	96.93(95.9-97.7%)
(b) Global dataset								
Drug	TP	FP	TN	FN	VME (95% CI)	ME (95% CI)	PPV (95% CI)	NPV (95% CI)
Ethambutol	1381	449	3484	187	11.93(10.4-13.6%)	11.42(10.5-12.4%)	75.46(73.4-77.4%)	94.91(94.1-95.6%)
Isoniazid	2781	54	2704	184	6.21(5.4-7.1%)	1.96(1.5-2.5%)	98.1(97.5-98.5%)	93.63(92.7-94.5%)
Pyrazinamide	750	148	2437	264	26.04(23.4-28.8%)	5.73(4.9-6.7%)	83.52(81.0-85.8%)	90.23(89.0-91.3%)
Rifampicin	2634	60	2990	136	4.91(4.2-5.8%)	1.97(1.5-2.5%)	97.77(97.1-98.3%)	95.65(94.9-96.3%)
Amikacin	100	4	1496	21	17.36(11.6-25.1%)	0.27(0.1-0.7%)	96.15(90.5-98.5%)	98.62(97.9-99.1%)
Capreomycin	97	25	1601	42	30.22(23.2-38.3%)	1.54(1.0-2.3%)	79.51(71.5-85.7%)	97.44(96.6-98.1%)
Ciprofloxacin	1	4	33	4	80.0(37.6-96.4%)	10.81(4.3-24.7%)	20.0(3.6-62.4%)	89.19(75.3-95.7%)
Kanamycin	88	3	1410	47	34.81(27.3-43.2%)	0.21(0.1-0.6%)	96.7(90.8-98.9%)	96.77(95.7-97.6%)
Moxifloxacin	14	1	85	3	17.65(6.2-41.0%)	1.16(0.2-6.3%)	93.33(70.2-98.8%)	96.59(90.5-98.8%)
Ofloxacin	329	19	1348	59	15.21(12.0-19.1%)	1.39(0.9-2.2%)	94.54(91.6-96.5%)	95.81(94.6-96.7%)
Streptomycin	462	27	2281	217	31.96(28.6-35.6%)	1.17(0.8-1.7%)	94.48(92.1-96.2%)	91.31(90.1-92.4%)
(c) Prospective dataset								
Drug	TP	FP	TN	FN	VME (95% CI)	ME (95% CI)	PPV (95% CI)	NPV (95% CI)
Ethambutol	71	49	4170	1	1.39(0.2-7.5%)	1.16(0.9-1.5%)	59.17(50.2-67.5%)	99.98(99.9-100.0%)
Isoniazid	312	19	3931	17	5.17(3.3-8.1%)	0.48(0.3-0.7%)	94.26(91.2-96.3%)	99.57(99.3-99.7%)
Pyrazinamide	102	22	4048	23	18.4(12.6-26.1%)	0.54(0.4-0.8%)	82.26(74.6-88.0%)	99.44(99.2-99.6%)
Rifampicin	133	32	4143	0	0.0(0.0-2.8%)	0.77(0.5-1.1%)	80.61(73.9-85.9%)	100.0(99.9-100.0%)
Amikacin	3	0	59	1	25.0(4.6-69.9%)	0.0(0.0-6.1%)	100.0(43.9-100.0%)	98.33(91.2-99.7%)
Capreomycin	3	0	59	0	0.0(0.0-56.1%)	0.0(0.0-6.1%)	100.0(43.9-100.0%)	100.0(93.9-100.0%)
Ciprofloxacin	34	3	199	15	30.61(19.5-44.5%)	1.49(0.5-4.3%)	91.89(78.7-97.2%)	92.99(88.8-95.7%)
Kanamycin	4	0	54	0	0.0(0.0-49.0%)	0.0(0.0-6.6%)	100.0(51.0-100.0%)	100.0(93.4-100.0%)
Moxifloxacin	19	3	41	1	5.0(0.9-23.6%)	6.82(2.3-18.2%)	86.36(66.7-95.2%)	97.62(87.7-99.6%)
Ofloxacin	21	3	42	1	4.55(0.8-21.8%)	6.67(2.3-17.8%)	87.5(69.0-95.7%)	97.67(87.9-99.6%)
Streptomycin	36	16	685	21	36.84(25.5-49.8%)	2.28(1.4-3.7%)	69.23(55.7-80.1%)	97.03(95.5-98.0%)

### Performance evaluation on Oxford Nanopore Technologies data

We evaluated *Mykrobe* performance on five samples where we had both Oxford Nanopore Technologies (ONT) and Illumina sequence data. The resistance calls made by *Mykrobe* on the Illumina and ONT data were in complete agreement, with the same variant calls made on each sample and therefore the same resistance profiles. Three of the samples were susceptible to all drugs (ERS3036287, ERS3036289, and ERS3036290), one sample (ERS3036288) was resistant to isoniazid, and the fifth sample (ERS3036286) was resistant to seven of the 11 drugs called by *Mykrobe* ( *Source data* ont.tsv) ^37^.

### Minor alleles improve prediction

Phenotypic tests by the indirect proportion method are defined in such a way as to call an isolate resistant if more than some proportion *P* of bacilli are resistant ^38^ - typically *P* = 0.01. Whole genome sequencing to a depth of 30-50x after several weeks of culture is by comparison relatively insensitive to minor populations. If we exclude for a moment the issues of clonal interference between resistant alleles, and selection due to culture, any single minor resistant allele detectable by sequencing should easily be detectable by phenotyping. This is essentially the motivation for *Mykrobe* using minor alleles to predict resistance. To do this, instead of specifying a minimum frequency threshold to detect minor alleles, *Mykrobe* performs a likelihood comparison between two statistical models: first, coverage on minor allele due to sequencing error (at rate around 0.01) and second, coverage on minor allele due to a minor population at frequency 0.2. In practise, with coverage around 30-50x, this results in a minor population being detected when has frequency above around 8-10%.

Having made minor changes to the catalogue from CP1 to CP2, we measured performance on the Global dataset, to finalise catalog and parameters (described below) before evaluating on the (final) Prospective dataset. The one remaining parameter that could be changed was the frequency of minor allele in the second model above, set to 0.2. We measured the impact of resistance calls driven by minor alleles using CP2 on the Global set - results are shown in Table 3. These results are broadly in concordance with those reported in 1 on the Training set. For second line drugs, the use of minor variants increases sensitivity without impacting false positive rate. In fact for amikacin, kanamycin, ofloxacin and streptomycin there were no false positive calls at all, and the proportion of resistance which was explained by minor alleles varied from around 1%-10%. For rifampicin and isoniazid, although the net contribution was positive, the error rate was slightly higher (8.3% and 6.7% of minor-allele-driven resistance calls were false). For ethambutol and pyrazinamide, however, although there were 29 and 45 true resistant calls made due to minor alleles, the proportion of false positive minor-driven resistant calls was even higher: 38% and 15%, respectively. If we compare the error rates in minor-allele-driven resistance calls for first-line drugs (6.7%, 8.3%, 38.3, 15.0% for rifampicin, isoniazid, ethambutol, pyrazinamide, see Table 3) with the overall rates driven primarily by major-allele calls (2.0%, 2.0%, 11.0%, 6.9%, see Table 1b) we see the relative differences between drugs are preserved - indeed the minor-allele-driven rates are all 3-4x the overall rates. Thus, the catalogue drives some or much of the between-drug differences. There are several potential explanations for the higher error rates for minor-allele calls. First, mutations that cause a low-level increase in MIC (minimum inhibitory concentration) may not cause a resistant phenotype if in a minor population - this is particularly likely for ethambutol where most mutations only increase the MIC slightly. Second, for pyrazinamide the phenotypic test is defined differently to most other drugs - the critical proportion *P* at which resistance is detected is 0.1 rather than 0.01 as for other drugs ^38^. In other words, the pyrazinamide phenotypic test is less sensitive to low frequency populations than those for other drugs.

**Table 3.** Minority variant calls made by *Mykrobe* using CP2 on the Global dataset. The second column shows the number of samples where the resistance phenotype is known for the given drug. Column 4, headed "Minor R calls" shows the number of resistant calls due to a minor population resistance allele. Column 5, headed "Error rate of minor R calls", shows the percentage of minor-allele-driven resistant calls that were incorrect versus phenotype.

Drug	Samples	Resistant samples	Minor R calls	Error rate of minor R calls (%)
Ethambutol	5501	1568	47	38.3
Isoniazid	5723	2965	24	8.33
Pyrazinamide	3599	1014	53	15.09
Rifampicin	5820	2770	45	6.67
Amikacin	1621	121	9	0.0
Capreomycin	1765	139	10	10.0
Kanamycin	1548	135	9	0.0
Ofloxacin	1755	388	37	0.0
Streptomycin	2987	679	10	0.0

In contrast to the above, for second-line drugs the pattern is different, with *lower* error rates for minor-driven calls. The number of samples with second-line phenotypes is too low to draw strong conclusions. We would like to see these results replicated with comprehensive phenotyping and deep sequencing.

In theory the *Mykrobe* statistical test described above could have been modified to use a second model with higher minor-allele frequency than the current value of 0.2 - essentially demanding a higher frequency to reduce false positives. Indeed, it might be possible to fit different thresholds per drug, improving results on our dataset. However, we had concerns about generalisability. A recent publication ^39^ showed how sequencing the same isolate with Illumina HiSeq, MiSeq and NextSeq could give different pictures of minor allele variation, including very different minor allele frequencies. We therefore elected to leave the model unchanged.

### Evaluating policy of ignoring unknown mutations in resistance genes

A key component of the approach taken by the CRyPTIC consortium in 20 was to refuse to make predictions if an unknown mutation was detected in a resistance-associated gene, and divert such samples to phenotyping. By contrast, *Mykrobe* genotypes known SNPs and indels, but does not detect novel mutations, and makes predictions of phenotypes based on these data only. In particular, if no resistance mutation from its catalogue is detected, *Mykrobe* makes a Susceptible prediction. Our prior expectation was that this would come at the cost of a higher false susceptible rate for *Mykrobe*. This was indeed the case - see the red components of the bars on the right hand side of Figure 3. However, in addition, on both the Global and Prospective datasets, we find that *Mykrobe* calls more true positives and negatives (i.e. correct resistant and susceptible calls) - see the grey segments of bars on Figure 3 (in particular on the left) where the CRyPTIC approach avoids making a call. The results (see Table 4) are consistent across the four first-line drugs: on the Global set *Mykrobe* misses around 60-170 resistant samples that the CRyPTIC process theoretically detects by reverting to phenotyping (with the caveat that phenotypic assays are themselves not 100% reliable). Although this is accompanied by more rapid detection of 100-300 more susceptible samples (which CRyPTIC detects later, by phenotyping), this provides strong motivation to add the ability to detect novel variants to *Mykrobe* in future releases. We note that this functionality is also missing from the other tools we benchmarked against, and that all of them could add this. Finally, we recall again that the global set is a heterogeneous combination of datasets sampled in different ways. The prospective set is prospectively sampled, though from low-burden countries in Europe. On that dataset we find the *Mykrobe* approach pays far less penalty, missing 0, 9, 2 and 17 samples resistant to rifampicin, isoniazid, ethambutol and pyrazinamide, respectively, while detecting 215, 184, 486, 76 susceptible samples earlier (without phenotyping).

**Table 4.** Comparing the impact of no-calls when an unknown mutation is detected (process used in 20) versus that of *Mykrobe*.

Global dataset				
Tool	Drug	TN	TP	FN
Mykrobe	Rifampicin	**2990**	**2634**	136
NEJM-2018	Rifampicin	2833	2614	**72**
Mykrobe	Isoniazid	**2704**	**2781**	187
NEJM-2018	Isoniazid	2564	2747	**86**
Mykrobe	Ethambutol	**3484**	**1381**	187
NEJM-2018	Ethambutol	3149	1339	**82**
Mykrobe	Pyrazinamide	**2437**	**750**	264
NEJM-2018	Pyrazinamide	2176	732	**90**
Prospective dataset				
Mykrobe	Rifampicin	**4143**	**133**	0
NEJM-2018	Rifampicin	3928	124	0
Mykrobe	Isoniazid	**3931**	**312**	17
NEJM-2018	Isoniazid	3747	311	**8**
Mykrobe	Ethambutol	**4170**	71	49
NEJM-2018	Ethambutol	3684	71	**47**
Mykrobe	Pyrazinamide	**4048**	102	23
NEJM-2018	Pyrazinamide	3972	**109**	**6**

### Benchmarking against other tools

In order to separate the effect of mutation catalogue and genotyping method, we gave ARIBA the same catalogue (namely the final release panel described above) as *Mykrobe* (with small differences, see *Methods* for details). For the other tools, we simply used their default catalogues in their latest versions at the time of benchmarking. The sensitivity, specificity, and error rates for *Mykrobe*, ARIBA, KvarQ, MTBseq, and TB-Profiler when applied to the Prospective dataset, summarised across all drugs, is given in Table 5 (full data are in the *Extended data* file accuracy_stats.tsv ^33^).

**Table 5.** Accuracy statistics of each tool and each dataset, using all available drugs. All values are percentages. The best value for each statistic is shown in bold. Drugs used for each dataset are as follows. Training: Amikacin, Capreomycin, Ciprofloxacin, Ethambutol, Isoniazid, Kanamycin, Moxifloxacin, Ofloxacin, Rifampicin, Streptomycin; Global and Prospective: Amikacin, Capreomycin, Ciprofloxacin, Ethambutol, Isoniazid, Kanamycin, Moxifloxacin, Ofloxacin, Pyrazinamide, Rifampicin, Streptomycin. VME, very major error rate (false-negative rate); ME, major error rate (false-positive rate); PPV, positive predictive value; NPV, negative predictive value.

(a) Training dataset						
Tool	Sensitivity	Specificity	VME	ME	PPV	NPV
ARIBA	90.04	97.91	9.96	2.09	85.59	98.62
KvarQ	80.81	98.03	19.19	1.97	84.96	97.38
MTBseq	82.68	97.65	17.32	2.35	82.91	97.62
Mykrobe	**91.64**	**98.21**	**8.36**	**1.79**	**87.57**	**98.84**
TB-Profiler	89.71	95.02	10.29	4.98	71.25	98.53
(b) global dataset						
Tool	Sensitivity	Specificity	VME	ME	PPV	NPV
ARIBA	87.30	**96.22**	12.70	**3.78**	**91.64**	94.11
KvarQ	79.99	95.81	20.01	4.19	90.05	90.99
MTBseq	81.34	96.03	18.66	3.97	90.67	91.56
Mykrobe	**88.12**	96.16	**11.88**	3.84	91.58	**94.47**
TB-Profiler	87.39	92.89	12.61	7.11	85.36	93.95
(c) prospective dataset						
Tool	Sensitivity	Specificity	VME	ME	PPV	NPV
ARIBA	90.10	98.87	9.90	1.13	78.74	**99.54**
KvarQ	84.11	**99.21**	15.89	**0.79**	83.19	99.26
MTBseq	85.82	99.00	14.18	1.00	80.05	99.34
Mykrobe	**90.22**	99.16	**9.78**	0.84	**83.39**	**99.54**
TB-Profiler	83.01	97.24	16.99	2.76	58.33	99.19

On each of the three datasets, *Mykrobe* is the most sensitive, has the lowest very major error rate (false susceptible error rate), and the highest negative predictive value. *Mykrobe* has the best specificity, major error rate (false resistant rate) and positive predictive value on the Training dataset, and the best or second-best results for those statistics on the Global and Prospective datasets.

**Figure 5.**
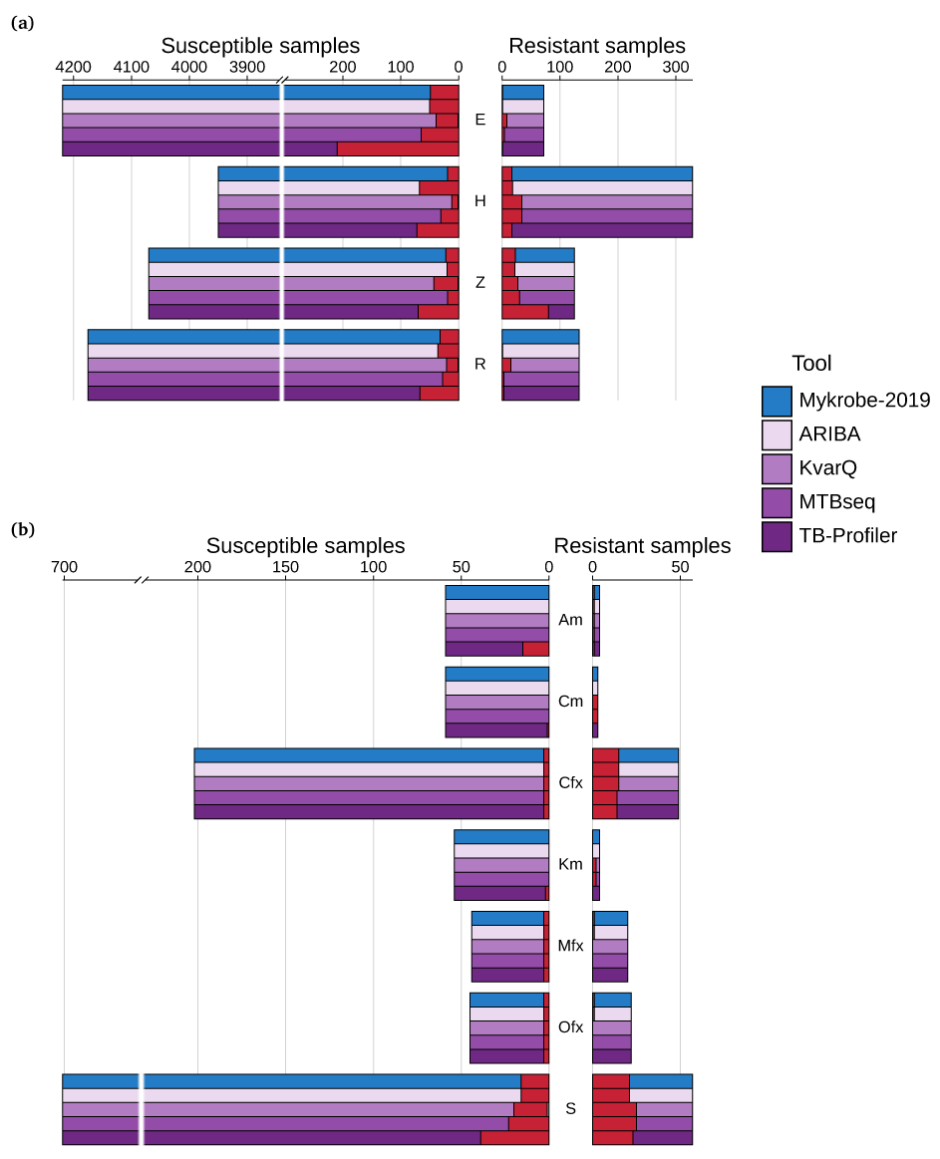
Comparison of ARIBA, KvarQ, MTBseq, *Mykrobe* (with final release panel) and TB-Profiler on the prospective dataset for ( **a**) first-line drugs and ( **b**) second-line drugs. Counts of susceptible samples are shown on the left, with true-negatives coloured by the tool/panel and false-positives coloured red. Similarly, resistant samples are shown on the right, with true-positives coloured by the tool/panel and false-negatives in red. E, ethambutol; H, isoniazid; Z, pyrazinamide; R, rifampicin; Am, amikacin; Cm, capreomycin; Cfx, ciprofloxacin; Km, kanamycin; Mfx, moxifloxacin; Ofx, ofloxacin; S, streptomycin.

A breakdown of the results on the Prospective dataset, showing each drug separately, is given in Figure 5. *Mykrobe* and ARIBA, as expected, are very similar except for the high false-positive rate for isoniazid for ARIBA, resulting in a PPV of 82% for ARIBA compared to 94% for *Mykrobe*. Manual inspection revealed that the extra incorrect calls were caused by assembly errors in the *katG* gene, resulting in false-positive calls because the gene appeared to be incomplete.

The memory usage and run time averaged across all runs of each tool is given in Table 6, and in *Extended data* files we have: summary boxplots in run_time_boxplots.pdf ^40^ and memory_boxplots.pdf ^41^, and the complete data in run_time_memory.tsv ^42^. ARIBA, KvarQ, *Mykrobe* and TB-Profiler all have low RAM usage (less than 1.1 GB), whereas MTBseq requires 12GB of RAM. KvarQ and MTBseq had the longest running time, taking on average 22 and 42 minutes respectively per sample. ARIBA, *Mykrobe* and TB-Profiler are fast, typically running in less than 5 minutes, with *Mykrobe* having the shortest median run time (3.2 minutes).

**Table 6.** Run time and peak RAM usage. Values for each tool are the median across all runs.

Tool	RAM (MB)	Time (m)
ARIBA	124	3.6
KvarQ	38	22.2
MTBseq	12201	41.6
Mykrobe	1057	3.2
TB-Profiler	863	4.5

### Impact of *Mykrobe* on initial choice of WHO-recommended regimen

The World Health Organisation (WHO) now recommends systematic access to drug susceptibility testing and specific TB treatment regimens based on individual drug resistance profiles. We therefore ask, if we were to base the choice of a patient’s initial multi-drug therapy purely on the genotyping results from *Mykrobe*, how accurate would this choice of treatment regimen be? We encoded the most recently published WHO TB treatment recommendations ( https://github.com/iqbal-lab-org/tb-amr-benchmarking/blob/master/python/evalrescallers/who_treatment.py), and used this to infer both the recommended drug regimen implied by the known phenotype of each sample, and that implied by *Mykrobe*’s resistance prediction (on the combined global and prospective datasets). The most recent WHO recommended regimens for MDR-TB involve a number of drugs for which phenotyping is rarely performed and for which the genetic basis of drug resistance is only partially understood (e.g. bedaquiline, linezolid). In these cases, drug administration and implementation of WHO recommendations in clinical contexts most often relies on the assumption of drug susceptibility. Given that, and the scarcity of available genotypic to phenotypic correlation data for those drugs, all isolates were treated as susceptible to bedaquiline and linezolid in this analysis. Also, given the absence of recent updated (2018) WHO recommendations for pre-XDR and XDR TB, this analysis model relies on previous recommendations ^24, 25^ which therefore limits the clinical significance of the analysis for the limited number of extensively resistant TB isolates. It also highlights the need for data, new guidance and subsequent update of the *Mykrobe* performance analysis for those most resistant isolates.

**Figure 6.**
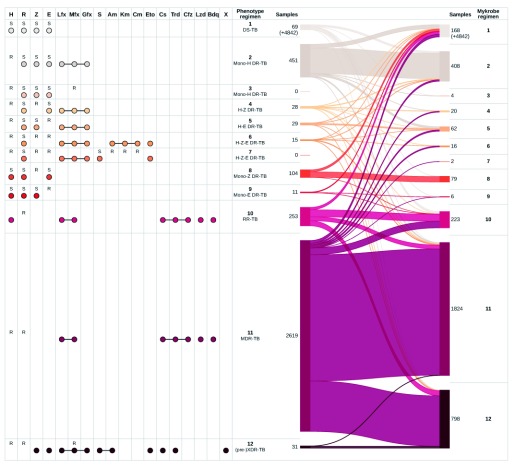
Comparison of drug regimen calls inferred from phenotype information and *Mykrobe* results, on the global and prospective datasets combined. See supplementary files regimen_plot.global.pdf, regimen_plot.prospective.pdf for the same plot for each of the global and prospective sets separately. On the left, “R” and “S” show the drug phenotypes used to identify the regimen. For example, resistance to isoniazid and moxifloxacin, and susceptibility to rifampicin, pyrazinamide and ethambutol implies drug regimen 3. Coloured dots show the drugs that are included in the regimen, where a line joining drugs represents interchangeability. The ribbons on the right show the mapping of phenotype-inferred regimens (left) to *Mykrobe*-inferred regimens (right), with numbers showing the number of samples allocated to each regimen. 4,842 samples called as regimen 1 by both methods are not shown. H, isoniazid; R, rifampicin; Z, pyrazinamide; E, ethambutol; Lfx, levofloxacin; Mfx, moxifloxacin; Gfx, gatifloxacin; S, streptomycin; Am, amikacin; Km, kanamycin; Cm, capreomycin; Eto, ethionamide; Cs, cycloserine; Trd, terizidone; Cfz, clofazimine; Lzd, linezolid; Bdq, bedaquiline; X, other WHO second-line drugs to which isolate is shown to be susceptible.

Figure 6 presents comprehensive resistance profiles, associated WHO-recommended regimen and a comparison of phenotype- and *Mykrobe*-driven therapies for all isolates. We immediately observe that for 98.6% (= 4842 */*(4842 + 69)) of phenotypically pan-susceptible isolates, *Mykrobe* would correctly imply first-line treatment (regimen 1 in the figure). We excluded those pan-susceptible isolates from the figure for increased clarity. Full results are available in *Extended data* file who_regimen_counts.tsv ^43^.

**Key error modes for Regimens 2-9** The most frequent clinically significant error mode was wrongly recommending a pan-susceptible TB regimen for mono-isoniazid resistant isolates, which occurred in 53 (12%) of the 451 mono-resistant (regimen 2) isolates. We describe below how all other benchmarked tools shared this error mode. Figure 6 also illustrates other, rarer, cases of non-MDR-TB and non-XDR-TB resistant isolates (regimens 3 to 9) for which a disagreement between phenotype-driven and *Mykrobe*-driven regimens results from undetected resistance (lines moving upwards as we go from left to right) or false-resistant genotyping results (line moving downwards as we go from left to right). Of the 187 isolates assigned to regimens 3–9 using phenotype data, *Mykrobe* suggested a pan-susceptible regimen (regimen 1) in 45 cases, and a RR-, MDR- or XDR-TB regimens (10, 11 or 12, respectively) in 3, 16 or 7 cases, respectively.

**RR-TB, MDR-TB and pre-XDR-TB regimens.** A significant number of discrepancies between phenotype-driven and *Mykrobe*-driven regimens revolve around regimen 10 (RR-TB), regimen 11 (the most recent WHO-recommended MDR-TB regimen), and regimen 12 (pre-XDR-TB) (see Figure 6 for regimen details). Regimens 10 and 11 only differ in that regimen 10 includes isoniazid, and is recommended if the isolate is known to be susceptible to that drug, or if the phenotype is unknown. Conversely, when comprehensive DST results are available, if they confirm a resistance profile including resistance to isoniazid, rifampicin a fluoroquinolone and/or an injectable aminoglycoside, a customized regimen including as many drugs to which the isolate is confirmed to be susceptible as possible is recommended. Given the limited number of XDR-TB isolates in our data, this analysis focused on quinolone resistant pre-XDR samples (regimen 12) for which we had sufficient data to recognise appropriate or erroneous treatment patterns. There were 2903 rifampicin resistant *M. tuberculosis* isolates for which first-line drug phenotyping justified initiation of RR-TB ( *N* = 253), MDR-TB ( *N* = 2619), or pre-XDR-TB ( *N* = 31) treatment regimen.

*Mykrobe* assigned 96 of the 253 phenotype-inferred RR-TB (regimen 10) isolates to that same regimen (8 of these had an unknown isoniazid phenotype). False-negative rifampicin calls by *Mykrobe* in 33 of the 253 (=13.0%) isolates resulted in a predicted pan-susceptible treatment (regimen 1). 54 of the 253 isolates were classified as MDR-TB (regimen 11) by *Mykrobe*, comprising 35 isolates with unknown isoniazid phenotype and 19 false-positive isoniazid calls. The remaining 70 isolates all had unknown phenotypes for isoniazid and moxifloxacin, whereas *Mykrobe* predicted resistance to these drugs and assigned them to XDR-TB regimen 12.

There were 2619 isolates classified as MDR-TB (regimen 11) from the phenotype data. The majority of isolates (1727/2619=65.9%) are appropriately assigned by *Mykrobe* to the MDR-TB regimen. A minority of isolates (103/2619=3.9%) are falsely identified as being less resistant and consequently directed (upwards in the figure) towards regimens 1–9. False-negative isoniazid calls resulted in 92 of the 2619 isolates (=3.5%) to be wrongly allocated to regimen 10 (RR-TB). The remaining 692 of the 2619 isolates (=26.4%) were identified as resistant to moxifloxacin, classifying them as regimen 12. Moxifloxacin phenotypic susceptibility testing results for 689 of these isolates was unknown, whereas *Mykrobe* determined them to be resistant and so directed the samples to a pre-XDR-TB regimen instead of an MDR-TB regimen. We can estimate what proportion of these 692 samples were correctly assigned to an XDR-TB regimen instead of to MDR-TB using the positive predictive value estimated from those samples with phenotypes (see Table 2). We estimate that for 644/692 (=93%) samples, the *Mykrobe*-driven regimen choice was accurate. We emphasise that this model relies on WHO recommendations which are expected to be updated in the specific case of pre-XDR-TB and XDR-TB.

**Comparison with other tools.** We find *Mykrobe* resulted in the correct regimen choice for 93.9% of samples using the latest panel, a 1.2% improvement over *Mykrobe* with the 2015 panel, and a 1% improvement over ARIBA using the same 2019 panel. We counted a tool as correct for RR-TB and MDR-TB isolates where phenotype data for isoniazid and/or moxifloxacin resistance was unknown, but the tool assigned MDR-TB or XDR-TB because of resistance calls for one or both of those drugs. All benchmarked tools performed reasonably similarly, with the worst performance by TB-profiler (89% correct). Per-tool accuracy of regimen choice is shown in Table 7, and Table 8, with the best-performing tool for each regimen highlighted. For 4 of the 11 regimens (numbers 1 (pan-susceptible), 2 (INH mono-resistant), 8 (pyrazinamide-mono resistant) and 10 (rifampicin-mono resistant)), *Mykrobe* achieves the highest success rate. In particular, the INH-mono-resistant case, highlighted above as a concern for *Mykrobe*, is also an issue for other tools.

**Table 7.** Benchmarking regimen success rates for different callers, on the global and prospective datasets combined.

Tool	Correct regimen	Incorrect regimen	Percent correct
Mykrobe-2019	7939	513	93.9
ARIBA	7853	599	92.9
Mykrobe-2015	7839	613	92.7
MTBseq	7770	682	91.9
KvarQ	7710	742	91.2
TB-Profiler	7519	933	89.0

**Table 8.** Regimen success rate broken down by regimen, on the global and prospective datasets combined. The regimens are: 1, DS-TB; 2, Mono-H DR-TB; 3, Mono-H DR-TB; 4, H-Z DR-TB; 5, H-E DR-TB; 6, H-Z-E DR-TB; 7, H-Z-E DR-TB; 8, Mono-Z DR-TB; 9, Mono-E DR-TB; 10, RR-TB; 11, MDR-TB; 12, pre/XDR-TB.

Regimen												
Tool	1	2	3	4	5	6	7	8	9	10	11	12
Mykrobe-2019	**98.6**	**77.4**	0	**21.4**	55.2	**6.7**	0	**72.1**	**9.1**	**79.4**	92.4	87.1
Mykrobe-2015	**98.6**	68.3	0	10.7	55.2	0.0	0	71.2	**9.1**	78.7	90.5	87.1
ARIBA	97.5	69.2	0	**21.4**	55.2	**6.7**	0	71.2	**9.1**	78.7	**92.7**	87.1
KvarQ	98.5	69.2	0	**21.4**	27.6	**6.7**	0	65.4	**9.1**	77.9	86.1	83.9
MTBseq	98.0	67.2	0	28.6	41.4	0.0	0	68.3	**9.1**	77.1	89.3	83.9
TB-Profiler	92.9	66.1	0	**21.4**	**58.6**	**6.7**	0	11.5	**9.1**	77.5	91.6	**90.3**

## Discussion

Although sequencing-based diagnostic information for *M. tuberculosis* has been available for some years ^1, 9, 11, 17, 44^, the results have not been sufficiently good to justify replacing (or partially replacing) phenotyping. However, very recently the CRyPTIC consortium showed that for first-line drugs, a prediction of susceptibility was now sufficiently accurate to support therapeutic management in routine clinical settings ^20^. Indeed this has been implemented now in Public Health England, RIVM (Netherlands) and the Wadsworth Centre (New York). In this study we introduce our reimplemented resistance prediction tool, *Mykrobe*, with initial mutation catalogue taken from that study ^20^ for first-line drugs, and 3 for second-line drugs. We evaluate *Mykrobe* in a number of ways. We show that the new catalogue does indeed give improved results over that from 3 used in our old implementation *Mykrobe predictor*, in particular increasing power to detect pyrazinamide resistance from 25% to 74% (global set, containing 1014 resistant samples) and 72% to 82% (prospective set, containing 125 resistant samples). This is achieved by considering all frameshifts of length 1 or 2 in *pncA* as causing pyrazinamide resistance, and comes at the cost of a increased major error rate (rising from 2.2% on the global set for the previous catalogue based on 3 to 5.7% for current *Mykrobe* (although there was no increase in major error rate in the prospective set, changing from 0.7% to 0.5%)). Given the poor performance of pyrazinamide phenotypic testing, some proportion of the discrepancies are probably due to phenotypic error ^45, 46^.

A key component in the approach of 20 was to flag novel mutations in resistance-associated genes, and refuse to make a prediction in those cases. In a putative clinical setting this would delay getting (conservative, correct) results until phenotypic DST was completed. By contrast *Mykrobe* does not do this, and predicts susceptibility when there is no mutation from the catalogue. We are able to compare and contrast these approaches, as we are using the same data, and conclude that the novel-mutation-aware approach is preferable to that used by *Mykrobe* (and, as far as we can tell, all other benchmarked tools). Essentially, novel (off-catalogue) mutations are an effective flag for very major errors (missed resistance). Our feeling is that the cost (delays for susceptible samples containing novel mutations) is outweighed by the benefit (avoiding some false susceptible calls). Modifying *Mykrobe* to follow that policy is therefore something we would like to do in the future. Of the other tools we tested, MTBSeq and TB-profiler would most easily be able to adopt this approach, as they both already do full SNP discovery for every sample.

In general *Mykrobe* outperforms all the other benchmarked tools, having the best sensitivity and best or second-best specificity on each of the three datasets. *Mykrobe* supports error-prone ONT reads, and we show that ONT and Illumina data produce identical results, although we would wish to have tested on more than five samples. Motivated by the fact that low frequency resistant populations will sweep to fixation under drug pressure, and by the results in the *Mykrobe predictor* paper ^1^, *Mykrobe* predicts resistance if it detects known resistance alleles at low frequencies (in practise above 10%, for illumina data only). For isoniazid, rifampicin and the second line drugs, this results in detection of more resistant isolates, at limited cost in false positives (see Table 3). However for ethambutol and pyrazinamide the proportion of these extra calls that are false is unacceptably high (38% and 15%, respectively). Possible reasons for different performance for these drugs are given above; future versions of *Mykrobe* are likely to exclude minor alleles for these drugs.

We introduce the idea of measuring how sequencing-based diagnostic for TB drug resistance impact potential regimen choice, and include a powerful way to visualise it. The idea is explicitly not to suggest this could directly advise physicians, but instead to provide a different metric for clinically important errors. Our analysis has limitations. First, we have only partial phenotyping data for second line TB drugs, and in fact none for the new and repurposed TB drugs. Second, WHO guidelines are ever evolving and may differ from clinical expert opinions when it comes to the choice of personalized therapeutic regimens. Nevertheless, we can clearly see that the richer resistance profile from WGS data leads to more differentiated choices of regimen. We look forward to impending large and fully phenotyped datasets, for example from the CRyPTIC consortium, where tens of thousands of global samples will have quantitative phenotype data for 14 drugs. In addition, CRyPTIC is set up to deliver improved resistance catalogues, which we intend to incorporate into *Mykrobe*.

The pipelines for running the analyses in this paper are fully automated, all the way to generating figures, tables of results and *Extended data*, and we intend to update the paper as results improve (and as competitor tools are updated; a future update will benchmark the latest TB-profiler release, which appeared during the preparation of this paper). Our recommendations for *in silico* DST tools such as *Mykrobe* reflect our own learnings: use minor populations to improve predictions, flag novel mutations in resistance genes, and allow user-defined catalogues to facilitate comparisons and testing.

## Conclusions

Antibiotic resistance prediction is of critical importance for the roll-out of sequencing-based diagnostics for TB. We have demonstrated here our new tool *Mykrobe*, supporting both nanopore and Illumina data. *Mykrobe* provides simple, automated and lightweight results which we evaluate thoroughly on over 10,000 isolates. We find that *Mykrobe* outperforms other tools, and (by implementing our catalogue in a second tool, ARIBA) confirm that the primary determinant of success is the resistance catalogue. We find that considering minor populations, and flagging novel (off catalogue) mutations in resistance genes improve results. Finally, we introduce a new metric to highlight errors which impact initial choice of therapeutic regimen, achieving an accuracy of 95%.

The field has further to go before we can say we are fully fit for purpose, especially for the second-line, novel and repurposed drugs. A genomic resistance predictor must by necessity be a living and growing project, and our intention is that this document should also be updated in parallel, providing evidence and also open underlying data. We will be updating it to include benchmarking of future panels of *Mykrobe*, and updated versions of other tools.

## Methods

### Implementation

***Species identification***. A hierarchy of sequence probes are used to identify species and “phylogroup” (in this case Non Tuberculous Mycobacteria (NTM)) or *Mycobacterium tuberculosis* Complex (MTBC)). This is done by building a de Bruijn graph of a training set of samples of known species, identifying which species each unitig (maximal non-branching path) is seen in, and then selecting probes (unitigs) which are highly differentiated between species, and between phylogroups. The method was previously described in Bradley *et al.* ^1^.

***Lineage identification with MTBC***. *Mykrobe* currently uses the lineage-informative SNPs from 47 to assign lineage within the MTBC.

***New statistical model for genotyping***. In order to use a de Bruijn graph to genotype a catalogue of variants (SNPs and indels) we first convert the list of variant sites into a set of sequence ‘probes’ of length 2 *k −* 1. If the resistance mutations are defined in amino acid space, we first convert these into a set of possible codon changes in DNA space. In the simple case, we then represent these alleles as two short sequences (of length 2 *k −* 1) - one “reference” or “susceptible” allele, and the other “alternate” or “resistant” allele. However, if there is another SNP or indel within *k* bases, any sample with this variant would have different susceptible or resistant allele *k*-mers. We therefore build equivalent susceptible or resistant alleles for each variant within *k* bases of our SNP of interest. These lead to at least two alleles for each SNP, and several more if there is background variation within *k* bases. To construct a catalogue of these background variants, we used all SNPs and indels called in 200 samples selected at random from the Training set.

From this set of probes, we construct our reference de Bruijn graph. Given an input FASTQ file, the de Bruijn graph is created, and intersected with this reference graph. For each probe allele we calculate the proportion of *k*-mers with non-zero *k*-mer coverage, and the median *k*-mer depth on each probe. For all mutations in the panel we iterate through all possible nucleotide changes that would generate the specified amino acid change (if appropriate), and find the resistant allele and susceptible allele with the highest coverage. We then compare three competing *k*-mer producing Poisson models: homozygous reference (‘0/0’), heterozygous (‘0/1’) (frequency=50%), and homozygous alternate (‘1/1’) specified as follows

0/0:

KmerCount(ref)∼Pois(D(1-ε)),

$$\text{KmerCount}(\text{alt})∼\text{Pois}\left(\frac{D\varepsilon }{3}\right)\text{;}$$

1/1:

$$\text{KmerCount}(\text{ref})∼\text{Pois}\left(\frac{D\varepsilon }{3}\right)\text{,}$$

KmerCount(alt)∼Pois(D(1-ε));

0/1:

$$\text{KmerCount}(\text{ref})∼\text{Pois}\left(\frac{D}{2}+\frac{D}{2}\frac{\varepsilon }{3}\right),$$

$$\text{KmerCount}(\text{alt})∼\text{Pois}\left(\frac{D}{2}+\frac{D}{2}\frac{\varepsilon }{3}\right);$$

where KmerCount() is a function returning the number of *k*-mers observed from a given allele (i.e., the sum of the *k*-mer coverages along an allele), *L* is the number of *k*-mers in that allele, *D* is the genome-wide average depth of coverage, which corresponds to an expected *k*-mer coverage of *D′* = *D*( *R − k* + 1) */R*, ** is the expected *k*-mer count along the allele ( ** = *LD′* ), and *ε* is the expected error rate. The log-likelihood of each allele is summed, and the maximum likelihood model is chosen. The confidence is given by the difference between the log-likelihoods of the two most likely models.

The following filters are applied to the resulting genotype calls resulting in a NULL call for the variant:

- LOW_GT_CONF: If the confidence of the call is below a certain value (default 150 for Illumina, dynamically assigned for nanopore)
- LOW_PERCENT_COVERAGE: If proportion of *k*-mers on the allele with non-zero coverage is not 100%
- LOW_TOTAL_DEPTH: If the total depth on a variant (reference + alternate) is less than min_proportion_expected_depth (default 0.30) of expected depth.

***Oxford Nanopore evaluation***. For Oxford nanopore data, which has a much higher per-base error rate than Illumina, the LOW_GT_CONF filter default value is determined empirically from the input data, as follows. First, the SNPs/indels are genotyped as described above using a default error rate of 15%. Restricting to variants called as homozygous (and assuming these calls are correct), the rate of error *k*-mers appearing on the wrong allele is estimated by dividing the total *k*-mer depth on all the non-called alleles by the total *k*-mer depth seen across both alleles in all calls. Next, the coverages on 10,000 SNPs are simulated as follows: for each SNP sample the depth on the "true" allele from a Poisson distribution with mean equal to the mean depth, and the depth on the "false" allele by sampling from a binomial distribution, with the number of trials equal to the mean depth, and probability equal to the estimated incorrect *k*-mer error rate. The genotype confidence of each SNP is calculated, and together these 10,000 simulated SNPs give us a modelled genotype confidence distribution. We then choose the genotype confidence cutoff to be the value that would retain 90% of the simulated SNP calls (this is the default - the precise value can be changed by the user). Genotyping is then re-run with the estimated *k*-mer error rate and this GT_CONF threshold.

### Operation

*Mykrobe* is available for Linux (command-line only), Mac (command-line or graphical user-interface (GUI) application) and Windows (GUI only). For Linux, Python 2 or 3 (minimum versions 2.7 or 3.4+) is required to install and run the software.

***Command-line version***. Installation of the command line version is via cloning the git repository and running some install commands (documented here: https://github.com/Mykrobe-tools/mykrobe), or by using bioconda:


conda install -c bioconda mykrobe


Drug resistance predictions can be obtained from a sample using the single command of the form:


mykrobe predict --format json --seq reads.fastq --output out.json sample_name species


where “sample_name” can be replaced with the name of the sample being run (any text will do). The “species” parameter can be “tb” or “staph” to use the built-in panels, or “custom” for a user-generated panel (an example is shown later). The output file “out.json” contains variant and drug resistance calls, and details of the run, such as the version number of *Mykrobe* and input parameters. It is in the standard JSON format, which can be easily parsed by standard libraries in most programming languages. Below is an example excerpt of the “susceptibility” section of the output, which contains resistance calls.


"susceptibility": {
    "Isoniazid": {"predict": "S"},
    "Rifampicin": {
        "predict": "R",
        "called_by": {
            "rpoB_I491F-ATC761277TTC": {
                "genotype": [1,1],
                "genotype_likelihoods": [
                    -18289.715541997128,
                    -99999999,
                    -120.40490302280631
                ],
                "info": {
                    "coverage": {
                        "reference": {
                            "percent_coverage": 0.0,
                            "median_depth": 0,
                            "min_non_zero_depth": 0,
                            "kmer_count": 0,
                            "klen": 21
                        },
                        "alternate": {
                            "percent_coverage": 100.0,
                            "median_depth": 150,
                            "min_non_zero_depth": 148,
                            "kmer_count": 3010,
                            "klen": 21
                        }
                    },
                    "expected_depths": [
                        145.0
                    ],
                    "conf": 18169
                }
            }
        }
    }
}


In the above example, the sample has been called as susceptible to Isoniazid ( "predict": "S") and resistant to rifampicin ( "predict": "R") because it has the variant I491F in the *rpoB* gene. The string ATC761277TTC tells us that this is a change from ATC to TTC at position 761277 of the reference genome H37Rv3. The various tag meanings are given in Table 9.

**Table 9.** JSON file tags.

Tag	Meaning
genotype	values 0/0, 0/1 or 1/1
genotype_likelihood	log likelihood of genotype. The closer to zero, the more likely it is.
percent_coverage	proportion of *k*-mers in allele present with *k*-mer coverage>0

Results can optionally be exported to a CSV file.


mykrobe predict --format csv --seq reads.fastq --output out.csv sample_name species


The above example would export as follows


sample     drug          susceptibility    called_by
1234       Isoniazid     S
1234       Rifampicin    R                 rpoB_I491F-ATC761277TTC:0:3010:18619


where the format of the called_by column is variant_name:reference_kmer_count:alt_kmer_count:conf.

***Using Mykrobe with user-defined panels for M. tuberculosis***. As described above, *Mykrobe* requires a set of sequence probes, which are generated from the list of variant sites. The panel of variant sites need to be in a tab-delimited file that has the gene name, variant, and whether the variant is a change in protein or DNA sequence. For example:


pncA     G12GC    DNA
gyrA     D94G     PROT
fabG1    T-8X     DNA


There are three variants in this example. The first is a change from G to GC at position 12 in the DNA sequence of the *pncA* gene. The second variant is an amino acid change from D to G at position 94 in the *embB* gene. The final variant is eight nucleotides upstream of the *fabG1* gene, where the T in the reference genome is changed to A, C, or G (i.e. X is a wildcard). Note that all positions are relative to the gene, not the complete genome sequence. The following command produces a FASTA file of probe sequences


mykrobe variants make-probes reference.fasta -g reference.gb -t panel.tsv > probes.fasta


where reference.fasta and reference.gb are FASTA and GenBank format files of the reference sequence and annotation respectively. Note that background variants can also be included in the probe set, which requires the variants in one or more VCF files, and MongoDB to be installed. The following BASH commands will add variants to a new database.


db=$PWD/mongo-db/
mkdir $db
mongod --quiet --dbpath $db &
sleep 10
for f in ‘cat vcfs.txt‘
do
    mykrobe variants add --db_name mtb $f reference.fasta
done


In the above commands, the file vcfs.txt contains a list of the VCF file names (one file name per line), and the variants from each VCF file are added in turn to the database. Then the same command as above can be run to make the probe sequences, but with the extra option --db_name mtb.

In addition to the probes FASTA file, a JSON file that maps each variant to a list of drugs is required to run the “predict” subcommand of *Mykrobe*. For example, the JSON file corresponding to the above example three variants is as follows.


{"pncA_G12GC": ["Pyrazinamide"],
 "gyrA_D94G": ["Ciprofloxacin", "Ofloxacin", "Moxifloxacin"],
 "fabG1_T-8X": ["Isoniazid"]}


The probes FASTA file probes.fasta and JSON file of variant to drug(s) variants.json can be used with *Mykrobe* with the command


mykrobe predict --seq reads.fastq --output out.json --panel custom \
  --custom_probe_set_path probes.fasta --custom_variant_to_resistance_json variants.json \
  <any sample name> tb


**Figure 7.**
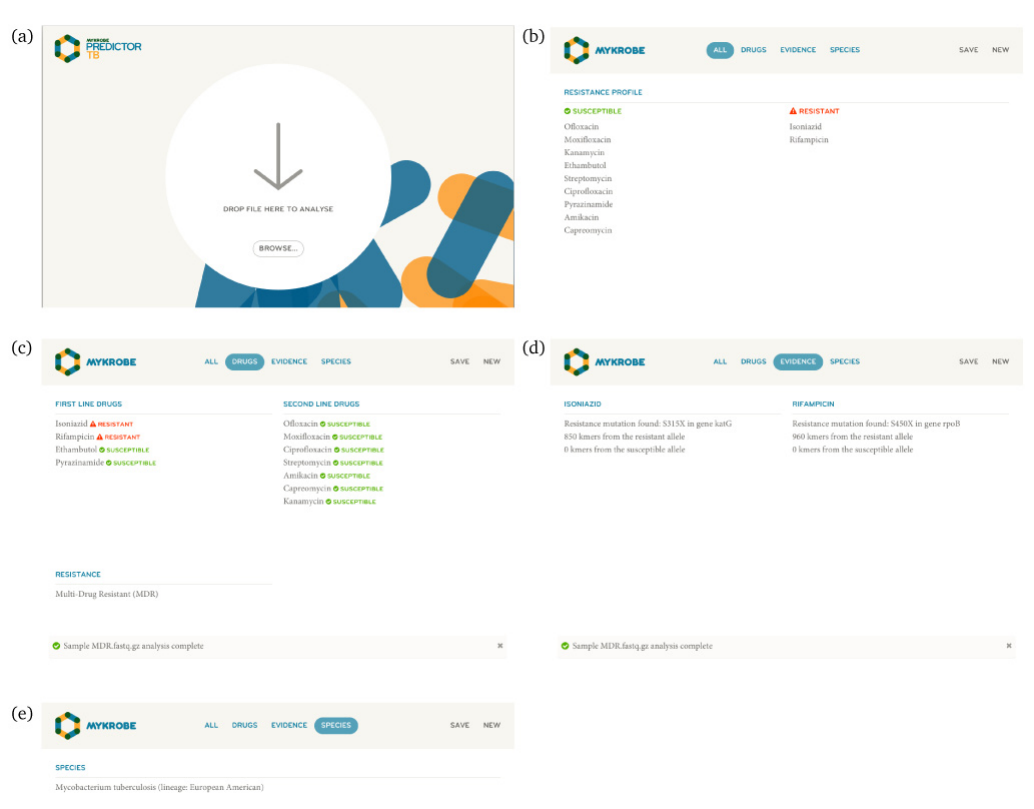
( **a**) Start page of the *Mykrobe* application. Users can drag-and-drop or select their sequence files. Once selected, *Mykrobe* starts the analysis process. ( **b**) Once the analysis is complete, the user is shown the resistance profile - which drugs is the isolate predicted to be resistant to, and susceptible to. ( **c**) Resistance profile can be broken down into first and second line drugs. ( **d**) The identified genetic substrate for resistance prediction can be seen in the "Evidence" tab. Here, evidence for isoniazid and rifampicin resistance is observed in variants *KatG* position 315 and *rpoB* position 450, respectively. ( **e**) The species and lineage prediction can be seen in the "Species" tab. Here the sample is *Mycobacterium tuberculosis* of European/American lineage.

***Graphical user-interface app for Mac and Windows***. The interface for *Mykrobe* graphical app is shown in Figure 7.

***Containerised releases***. A Docker container can be obtained using the command


docker pull quay.io/biocontainers/mykrobe:0.7.0--py37h2666aa9_0


***Variant panel development***. The new *Mykrobe* variant catalogue was developed using the Training and Global datasets as follows (see also Figure 2). We started with the catalogue of variants from 20 for the first line drugs, which we call NEJM-2018. This was used together with the remaining drugs from the existing *Mykrobe predictor* release panel (Walker-2015), and the fluoroquinolones separated into ciprofloxacin, moxifloxacin and ofloxacin. This combined set of variant sites was called candidate panel 1 (CP1). Note that this includes all frameshifts of length 1 and 2 in *pncA* and *katG*, implying resistance to pyrazinamide and isoniazid respectively. *Mykrobe* was run on the Training dataset using CP1 and the results were compared against the known phenotypes for those samples. All variants that had positive predictive value less than 5% were removed from CP1, to make candidate panel 2 (CP2). Next, the process was repeated, removing variants from CP2 to make candidate panel 3 (CP3), based on the results of running *Mykrobe* with CP2 on the Global data set. All removed variants are provided in the *Source data* file removed_variants.tsv ^32^. Finally, three removed variants were reinstated into the panel because they were found to have positive predictive value greater than 5% on later datasets (these were identified using the results from the Walker-2015 panel). The final panel is included as the default set of variants in release 0.7.0 of *Mykrobe*.

***Comparison with other tools***. Since our combined Training, Global, and prospective datasets consist of more than ten thousand samples, we only benchmarked *Mykrobe* against tools which could be run on the command-line (not web or user-interface applications). Specifically, we tested ARIBA (version 2.13.2), KvarQ (commit d693f561d205c9a3f9b9c705e2fefecdeb715cc8), MTBseq (version 1.0.3) and TB-Profiler (commit 327e431c3e9de2897a885fabe6bfede1421b2470). In order to separate the impact of catalogue from the different genotyping processes applied by different methods, we modified ARIBA to use the same catalogue as *Mykrobe*, but for the other tools we used their in-built catalogues.

ARIBA was modified by adding an option to use the same variant panel as the final *Mykrobe* release panel, but with the following differences. First, since ARIBA performs local assembly, it was modified to call resistance to pyrazinamide or isoniazid if the *pncA* or *katG* genes respectively were not completely assembled without internal stop codons. This is different from the *Mykrobe* variant panel, which instead includes all nonsense mutations and indels of length 1 and 2 in those genes. Second, unlike *Mykrobe*, ARIBA was not set to report resistance-conferring synonymous changes in gene sequences. We note that ARIBA code version 2.13.2 was run for this study, but using the built-in panel from version 2.13.3, which corresponds to the final *Mykrobe* release panel. These versions only differ in that 2.13.2 has a variant panel corresponding to CP3.

*Mykrobe* and ARIBA explicitly have the fluoroquinolones ciprofloxacin, moxifloxacin, and ofloxacin in their variant catalogues. However, KvarQ, MTBseq and TB-Profiler simply call fluoroquinolone resistance without distinguishing between the three drugs. Therefore when reporting results, a fluoroquinolone call was taken to imply resistance to all three of ciprofloxacin, moxifloxacin, and ofloxacin.

The benchmarking pipeline was implemented using Nextflow ^48^, Singularity ^49^ and Python scripts, and is freely available under the MIT licence (code available here: https://github.com/iqbal-lab-org/tb-amr-benchmarking ; commit version used was 3e768c025ee54f154116dc6b2f7100a844081609). Running time and peak RAM usage of the tools was gathered using the output of the UNIX command time -v, using the fields “Elapsed (wall clock) time” and “Maximum resident set size”. The benchmarking pipeline outputs all data in a single JSON file (included as *Extended data* file pipeline.json.gz ^50^), which was then used to generate the results of this study, including all tables and figures, with the separate code repository (code here: https://github.com/iqbal-lab-org/tb-amr-benchmarking-paper/ ; the version/commit used to generate this paper was 59e1b442dd04ec8e16656334ab9ea7d5cefaa871). Singularity containers of the two repositories are available at https://doi.org/10.6084/m9.figshare.10623410.v1 and https://doi.org/10.6084/m9.figshare.10650515.v1.

***Oxford nanopore samples***. *Mykrobe* was evaluated on five samples sequenced using both Illumina and Oxford Nanopore Technologies (ONT) MinION (ENA project ERP113349; sample and run accessions are in additional file ont.tsv). Raw nanopore fast5 files were basecalled with ONT Albacore (version 2.1.7) using the command


read_fast5_basecaller.py --flowcell FLO-MIN106 --kit SQK-LSK108 --recursive --barcoding \
 --input data_in --output data_out --output_format fastq


Porechop (version 0.2.3) was then used to trim adapters from the reads and to do additional basecalling. When demultiplexing is done by Porechop on the Albacore demultiplexed folders, Porechop will assign reads to a barcode where both Albacore and it agree. All reads where there is disagreement between the two are placed in the “unclassified” category. The resulting FASTQ files for each barcode were aligned to the *M. tuberculosis* H37Rv reference (NC_000962.3 ^51^) using minimap2 ^52^ with the map-ont option. The resulting SAM file was sorted, indexed, and converted to FASTQ using SAMtools ^53^. When converting the SAM file to FASTQ, all unmapped reads (SAM flag 0x4) were filtered out. The final *Mykrobe* panel from release 0.7.0 was run on the samples, separately on each of the Illumina and ONT reads. For ONT reads, the option --ont was used to trigger the use of the dynamic genotype confidence cutoff, as described above.

### WHO regimen analysis

Using previously published WHO guidelines for treatment of drug susceptible and drug resistant *M. tuberculosis*, we established a set of rules to associate any given drug resistance profile to a recommended drug regimen ( *Extended data* file who_regimens.tsv ^54^ presents treatment rules) ^24, 25^. Regimens including multiple new drugs (such as bedaquiline and delamanid, where the basis of drug resistance is poorly understood and phenotyping is rare), were included in this model, but isolates were assumed to be susceptible, since phenotype data for these drugs was unavailable, and is not assumed to be available for the WHO protocols. ^26^.

## Data availability

### Source data

Figshare: sample_data.tsv. https://doi.org/10.6084/m9.figshare.7556789.v1 ^29^.

This project contains European Nucleotide Archive accession numbers for Illumina sequence data and associated metatdata.

Figshare: ont.tsv. https://doi.org/10.6084/m9.figshare.7605443.v1 ^37^.

This project contains European Nucleotide Archive accession numbers for ONT data, and matched illumina data, along with phenotypes and known resistance alleles.

Figshare: panel.CP1.tsv. https://doi.org/10.6084/m9.figshare.7605383.v1 ^30^

Figshare: panel.CP1.json. https://doi.org/10.6084/m9.figshare.7605416.v1 ^31^.

Figshare: panel.CP2.tsv. https://doi.org/10.6084/m9.figshare.7605386.v1 ^55^.

Figshare: panel.CP2.json. https://doi.org/10.6084/m9.figshare.7605422.v1 ^56^.

Figshare: panel.CP3.tsv. https://doi.org/10.6084/m9.figshare.7605389.v1 ^57^.

Figshare: panel.CP3.json. https://doi.org/10.6084/m9.figshare.7605425.v1 ^58^.

Figshare: panel.final.tsv. https://doi.org/10.6084/m9.figshare.7605395.v1 ^35^.

Figshare: panel.final.json. https://doi.org/10.6084/m9.figshare.7605428.v1 ^36^.

These files contain candidate panels 1, 2 and 3, and the final panel, in TSV and JSON file formats.

Figshare: removed_variants.tsv. https://doi.org/10.6084/m9.figshare.7605380.v1 ^32^

This file contains the variants removed from candidate panels 1 and 2 during development of the final variant panel.

Figshare: run_time_memory.tsv. https://doi.org/10.6084/m9.figshare.7605437.v1 ^42^.

This file contains the run time and peak memory usage of each tool on each sample.

### Extended data

Figshare: accuracy_stats.tsv. https://doi.org/10.6084/m9.figshare.7605398.v1 ^33^.

This file contains the summary statistics for each tool and drug.

Figshare: variant_counts.tsv. https://doi.org/10.6084/m9.figshare.7605407.v1 ^34^.

This file contains the number of true- and false-positive calls for each tool and each reported variant.

Figshare: run_time_boxplots.pdf. https://doi.org/10.6084/m9.figshare.7605431.v1 ^40^.

Figshare: memory_boxplots.pdf. https://doi.org/10.6084/m9.figshare.7605434.v1 ^41^.

These files contain boxplots summarising the run time of each tool, generated from the file run_time_and_memory.tsv.

Figshare: who_regimen_counts.tsv. https://doi.org/10.6084/m9.figshare.7851689.v1 ^43^.

This file contains WHO regimen counts for each tool and dataset.

Figshare: pipeline.json.gz. https://doi.org/10.6084/m9.figshare.7605509.v1 ^50^.

This file contains the otuput of the benchmarking pipeline.

Figshare: who_regimens.tsv. https://doi.org/10.6084/m9.figshare.7605476.v2 ^54^.

This file contains drug regimens defined by the WHO.

Data are available under the terms of the Creative Commons Zero "No rights reserved" data waiver (CC0 1.0 Public domain dedication).

## Software availability

Mykrobe is available at: http://mykrobe.com.

Source code available from: https://github.com/Mykrobe-tools/mykrobe.

Archived source code at time of publication: https://doi.org/10.5281/zenodo.3549926 ^23^.

License: MIT License.
