# Supplementary material for: Antibiotic resistance prediction for Mycobacterium tuberculosis from genome sequence data with Mykrobe
Source: Wellcome Open Res. 2019 Dec 2;4:191. [Version 1] doi: 10.12688/wellcomeopenres.15603.1 (PMC7004237; doi:10.12688/wellcomeopenres.15603.1)
Supplement: Peer review history

# Peer review: Reviewer response for version 1

**DOI:** 10.21956/wellcomeopenres.17090.r37677

**Published:** 2020-02-05

**Authors:** Conor J. Meehan

**Affiliations:** 1 School of Chemistry and Biosciences, University of Bradford, Bradford, UK

The article by Hunt *et al.* outlines the substantial improvements and validation of the new version of Mykrobe. The previous version was quite widely used and I think that the data and methods presented here will benefit the TB community greatly.

 I do not have any comments to be addressed outside of what the other two reviewers already raised. I think readability needs improving, including outlining the TB drug resistance problem more prominently in the introduction, clearly separating what was results from the previous version and what is new here and ensuring the methods, including dataset selection, are in the methods section as appropriate.

I confirm that I have read this submission and believe that I have an appropriate level of expertise to confirm that it is of an acceptable scientific standard.

# Peer review: Reviewer response for version 1

**DOI:** 10.21956/wellcomeopenres.17090.r37245

**Published:** 2020-01-29

**Authors:** Duncan MacCannell

**Affiliations:** 1 Office of Advanced Molecular Detection, National Center for Emerging and Zoonotic Infectious Diseases, Centers for Disease Control and Prevention, Atlanta, GA, USA

Thank you for the opportunity to review this manuscript from Hunt and colleagues on the development and validation of genotypic antibiotic resistance prediction for Mycobacterium tuberculosis using the open source Mykrobe platform. Building upon the existing Mykrobe Predictor platform, Mykrobe includes a number of important improvements for mycobacteriological analysis, including significant refactoring of the codebase, reengineering and algorithmic improvements, an updated catalog of functional susceptibility data and corresponding genotypic predictors, expansion to include new sequencing platforms, such as the ONT MinION, and renewed emphasis of user-centric and user-configurable design.

 Major comments:

- PAGE 5: The geographic breakdown of training, prospective and global sample subsets seems disproportionate. Presumably, this is due to the training and prospective datasets from specific sites having more reliable or complete data and functional susceptibility information? The multi-phased development and validation of the resistance prediction panel is otherwise well-described (text and Figure 2) and adequate, but the geographic sampling bias should at the very least be explained. PPV and NPV performance metrics were all impressive, with low overall error rate; critical discrepancies were adequately addressed or explained. 

Without delving into the primary data, the dataset tables on Page 10 using the final Mykrobe panel would seem to suggest significant underlying differences in PPV/NPV/VME/ME between datasets.

 This could be an issue if the characteristics of the three test populations used to develop and refine the final Mykrobe panel are not relatively homogenous.

 Minor comments:

1. PAGE 9: Interestingly (Figure 4), ciprofloxacin resistance false positivity seems to have increased slightly with Mykrobe-CP3, without corresponding shifts in the prediction of other fluoroquinolones.  This is noted on Page 9, which implicates D94A in gyrA, and discusses adjustments that were made to CP3 to accomodate. While I'm not by any means an expert in the specific mechanisms of fluoroquinolone resistance in TB, presumably Mfx and Ofx should also show some degree of impact? Seems odd.
2. PAGE 11-12: Assessing the impact of minor alleles and unknown mutations was a welcome addition to this report. Thank you.
3. PAGE 13-14: Comparative benchmarking with other software, using default databases and settings. Mykrobe performance appears to be most on-par with ARIBA (Page 13), albeit with significantly higher peak RAM utilization (Page 15). Summary tables from Page 13 and graphical breakdowns in Figure 5 may be slightly redundant and some of this could likely be condensed or moved to a supplemental.
4. The flow diagram on Page 16 is particularly useful in teasing apart the genotypic prediction and proposed drug regimen data. These data clearly underscore the difficulty in consistently assigning regimens from TB genotype data. (For example -- last paragraph, page 16: unless I'm misreading, Mykrobe genotype predictions missed a full 1/3 of calls for MDR-TB regimen). Even so, the overall performance (Table 7) shows a marked overall improvement over existing tools and previous iterations.

Overall - Very well done and an enjoyable manuscript to read, with clear improvements to the performance and execution of Mykrobe. Many thanks for allowing me to review.

I confirm that I have read this submission and believe that I have an appropriate level of expertise to confirm that it is of an acceptable scientific standard, however I have significant reservations, as outlined above.

# Peer review: Reviewer response for version 1

**DOI:** 10.21956/wellcomeopenres.17090.r37246

**Published:** 2019-12-19

**Authors:** Rebecca E. Colman

**Affiliations:** 1 Foundation for Innovative New Diagnostics (FIND), Geneva, Switzerland; 2 University of California, San Diego, CA, USA

Hunt and co-authors describe the improvements and testing of an antibiotic resistance prediction tool for *Mycobacterium tuberculosis* using NGS data. The authors clearly describe the improvements on the original *Mykrobe predictor* software resulting in this new version, *Mykrobe.* Improvements being: mutation catalog update, ability to use other mutation catalogs, improved identification of non-tuberculosis Mycobacterial species, and ability to use tool with Oxford Nanopore data. The software is freely available at mykrobe.com and has source code and documentation on Github. Additionally the data presented in the paper can be found on figshare. The development of the mutation panels and data interpretation is demonstrated on three datasets: training, prospective, and global set. The benchmarking against four other prediction tools is useful to show improvements and performance. Although only five Oxford Nanopore samples were used, they did show agreement in drug resistant variant calls. The case study of using *Mykrobe* predictions to guide personalized regimens is an extremely useful comparison and illustrates valuable clinical application of this type of tool.

 The manuscript is scientifically sound without further changes. However, the following minor comments should be considered to increase the readability of the manuscript.

 The paper is very lengthy and full of information, however it is hard to read and digest all the information contained within the manuscript. The abstract is concise and clear it would be beneficial for structure and format to be mirrored throughout the manuscript. For example, the introduction contains a lot of text dedicated to explaining the first version, *Mykrobe predictor,* and does not put the tool in context of tuberculosis until the fourth paragraph. Perhaps clearly outlining the need for this tool and then summarize how the original version has been improved will streamline the introduction and increase the impact of the work, as right now some of the benefits of the software are buried in a large amount of text.  

 Throughout the results section, there are portions that make more sense in the methods section, and then present on the results for only this study in the results section. For example, in the results species identification section, it is unclear which are the results for this manuscript versus previous findings. Additionally in the minor alleles prediction section, the rationale behind the work should be moved to the methods.

 In the results section, the table 1 does not include sensitivity/specificity as stated in the body. The data is there to calculate these values but the specific sensitivity and specificity values are not included. Additionally, the sensitivity and specificity stated in the abstract are not found elsewhere in the paper – they can be calculated with data given, but would be beneficial to have in results.

 The methods section should be organized to mirror the order of the results section as this would enable readers to quickly reference methods when reading the results section. In the methods variant panel development section, it is unclear what data sets CP2 and CP3 are run on to generate the next panels. This is clear in the figure 2, but text was unclear.

I confirm that I have read this submission and believe that I have an appropriate level of expertise to confirm that it is of an acceptable scientific standard.
